# Supplementary material for: Leveraging Deep Learning for Fine-Grained Categorization of Parkinson’s Disease Progression Levels through Analysis of Vocal Acoustic Patterns
Source: Bioengineering (Basel). 2024 Mar 21;11(3):295. doi: 10.3390/bioengineering11030295 (PMC10968564; doi:10.3390/bioengineering11030295)
Supplement: Supplementary file 1 [file bioengineering-11-00295-s001.zip › bioengineering-2927198-supplementary.pdf]

Supplementary Information

Leveraging Deep Learning for Fine-Grained Categorization of Parkinson's Disease Progression Levels through Analysis of Vocal Acoustic Patterns

Hadi Sedigh Malekroodi [1], Nuwan Madusanka [2], Byeong-il Lee [1,2,3,*] and Myunggi Yi [1,2,3,*]

[1] Industry 4.0 Convergence Bionics Engineering, Pukyong National University, Busan 48513, Republic of Korea; hadi_sedigh@pukyong.ac.kr
[2] Digital of Healthcare Research Center, Institute of Information Technology and Convergence,
Pukyong National University, Busan 48513, Republic of Korea; nuwanmadusanka@hotmail.com
[3] Division of Smart Healthcare, Pukyong National University, Busan 48513, Republic of Korea
* Correspondence: bilee@pknu.ac.kr (B.-i.L.); myunggi@pknu.ac.kr (M.Y.)

Supplementary materials include:

Table S1. Details of modified dataset**.**

Len. of audio post-seg.	Vowels	PD_early	PD_Adv.	HC	Total
FS1 and FS-5 (only first 1 or 5-sec)	/a/, /e/, /i/, /o/, /u/	160	115	200	475
	Each vowel by itself	32	23	40	95
AS-1 (1-sec segmentation)	/a/, /e/, /i/, /o/, /u/	3722	1817	3536	9075
	/a/	746	360	681	1787
	/e/	719	356	701	1776
	/i/	760	387	711	1858
	/o/	770	358	700	1828
	/u/	727	356	743	1826
AS-5 (5-sec segmentation)	/a/, /e/, /i/, /o/, /u/	529	244	473	1246
	/a/	103	49	89	241
	/e/	101	48	93	242
	/i/	112	52	96	260
	/o/	109	46	94	249
	/u/	104	49	101	254

Table S2. Comparison of performance (mean ± SD) with additional two recent models using the FS-5 datasets. The table compares precision, recall, F1-score, and accuracy across models**.**

**FS datasets**			**Models**				
		**Metric (%)**	**VGG16**	**VGG19**	**Dense121**	**Eff_b0**	**Swin_s**
5 sec	HC	Precision	96.67±4.71	96.67±4.71	96.67±4.71	96.67±4.71	**97.00±4.24**
		Recall	99.67±0.47	99.33±0.94	99±1.41	**100±0**	**100.00±0**
		F1 score	98.00±2.83	98.00±2.16	97.67±2.05	**98.33±2.36**	**98.33±2.36**
	PD_Mild	Precision	91.00±6.38	**92±3.56**	76±2.94	79.67±9.03	88.67±7.72
		Recall	82.67±8.06	73.00±3.74	79.33±16.44	79.33±6.6	74±22.45
		F1 score	**86**.00**±1.63**	81.00±3.56	76.67±9.29	79.67±7.76	77.67±12.5
	PD_Severe	Precision	84.67±8.96	75.33±4.5	78±12.73	76.33±8.38	77.67±13.27
		Recall	**88.67±9.74**	**92.33±3.09**	67.67±4.19	71.33±12.76	83.67±13.6
		F1 score	**85.67±5.91**	82.67±1.89	72±2.94	73.33±8.96	78.33±0.47
		Accuracy	**91.15±0.64**	88.84±1.54	84.8±44.09	86.09±8.22	87.39±3.92

AS-1 dataset after major voting (Accuracy [%])

VGG16

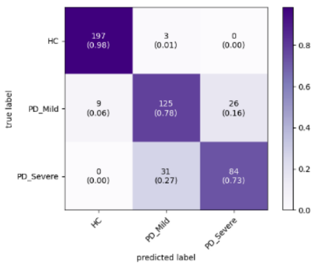

(85.47)

Resnet18

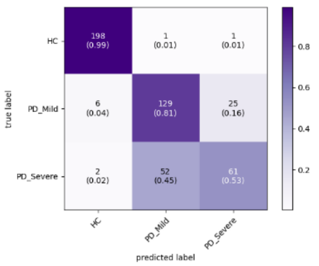

(81.68)

Swin_s

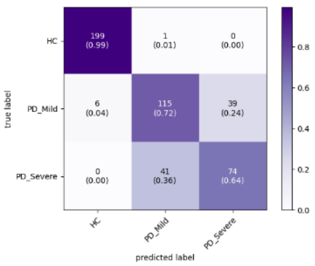

(81.68)

VGG19

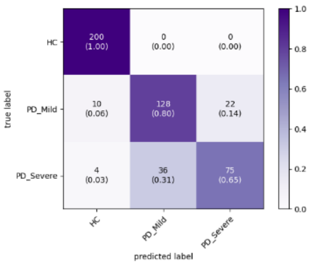

(84.48)

Resnet50

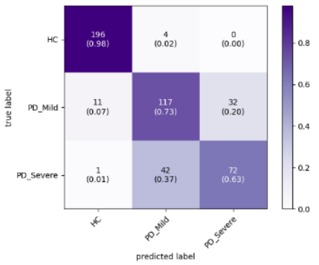

(81.05)

Swin_b

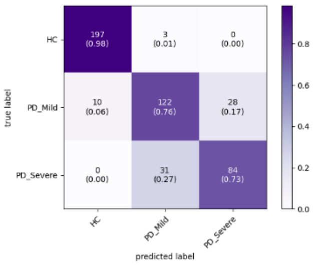

(84.84)

Figure S1. Cumulative confusion matrix for each model after applying majority voting to predictions on the AS-1 dataset.

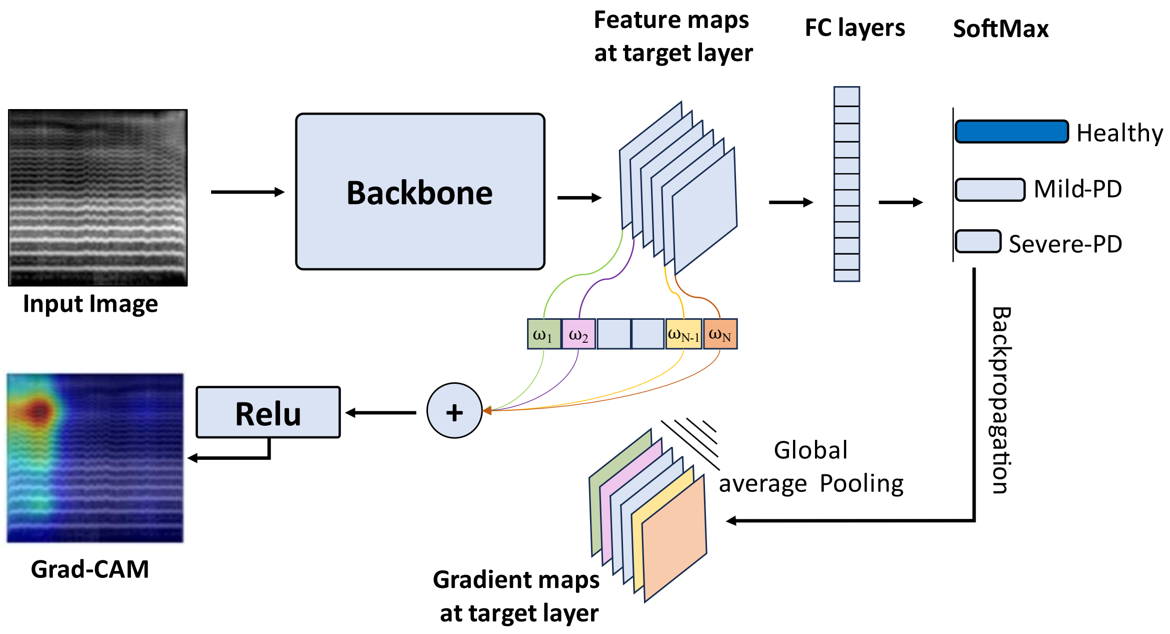

Figure S2. Architecture of Grad-CAM. An input image is fed through a trained convolutional neural network, which produces a classification result. Backpropagation is then performed to obtain the gradient of the classification score with respect to the feature maps of the last convolutional layer. The gradients are global-average-pooled to obtain weights that represent the importance of each feature map channel. The weighted combination of feature maps is passed through a ReLU activation to produce a coarse localization heatmap highlighting the relevant image regions for the predicted class [1]. Warmer colors like yellow, green, and especially red highlight regions that strongly activate the model in making its predictions. Meanwhile, cooler blue tones point to areas that have little effect on the model's reasoning. So, the overlay makes it possible to interpret which input regions contain features that are most contributory versus negligible for the model's inference on a given input image [1,2].

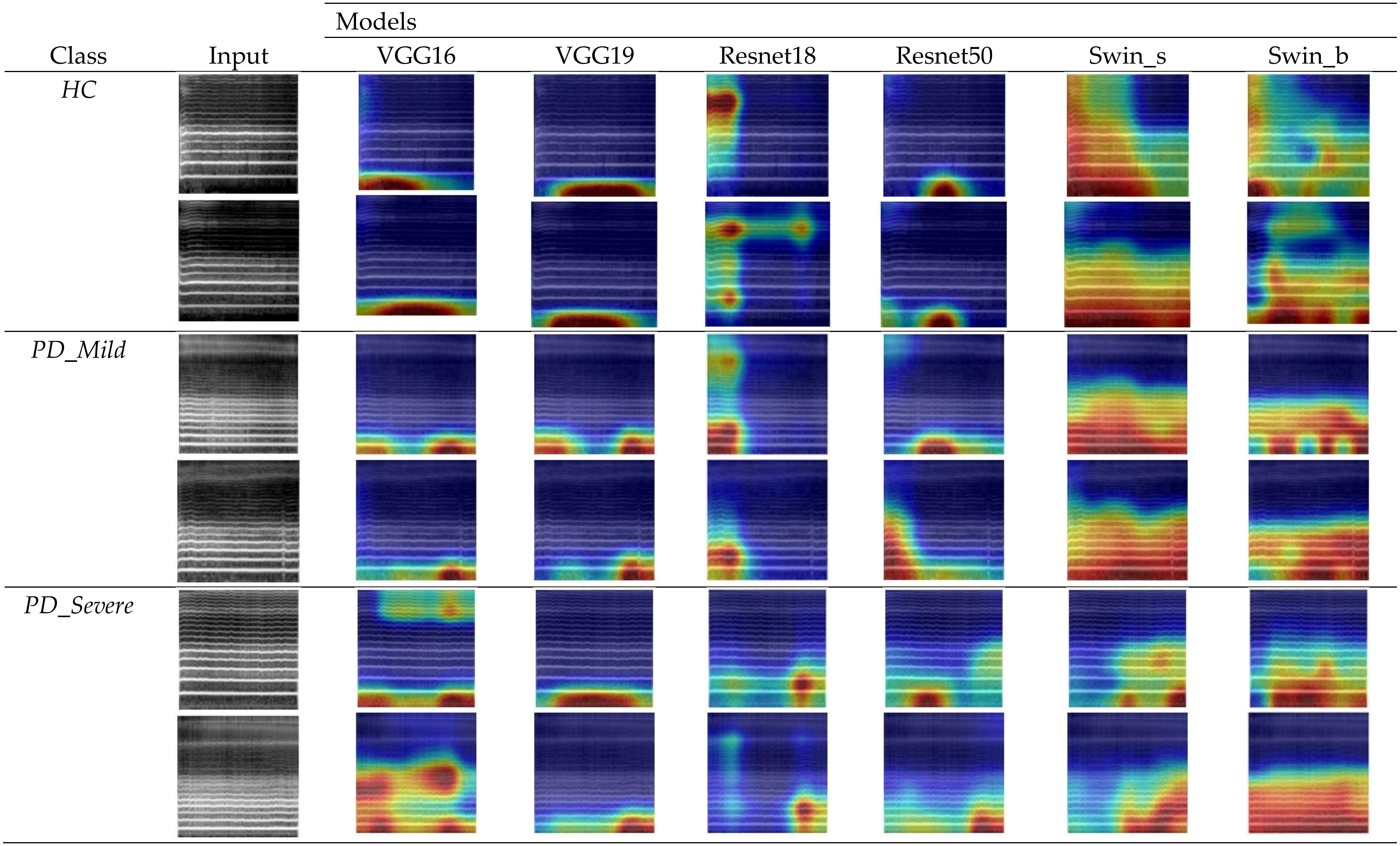

Figure S3. Grad-CAM visualization features different models across various classes for specific vowel /o/.

Reference

1. Mellak, Y., et al., *A machine learning framework for the quantification of experimental uveitis in murine OCT.* Biomedical Optics Express, 2023. **14**(7): p. 3413-3432.
2. Lal, K.N., *A lung sound recognition model to diagnoses the respiratory diseases by using transfer learning.* Multimedia Tools and Applications, 2023. **82**(23): p. 36615-36631.
